# Running for Boys

**Published:** 1909-04-03

**Authors:** 


### The Hospital

A Journal of

The Medical Sciences and Hospital Administration.

New Series. No. 109, Vol. V. [No. 1181, Vol. XLVI.] SATURDAY, APRIL 3, 1909.

### Running for Boys.

A short time ago, as will be remembered, a Mr. Farmer, who has been in his day a prominent footballer and athlete, created a good deal of stir by a letter to the Press in which he denounced the practice of permitting boys under nineteen years of age to take part in long-distance races—that is, of a mile or upwards. It will also be remembered that he backed up his views by producing the expression of a guarded but very similar view adopted by five highly eminent and well-known physicians and surgeons. A good deal of discussion ensued, but finally the matter dropped for the time being; it has, however, been revived by the publication of certain pronouncements by the Medical Officers of Schools Association. To these views great weight attaches. Of the special subject under consideration the members are better informed and better judges than specialists in consulting practice in London can possibly be, and the letter addressed by their president, Dr. C. E. Shelly, to the *Times* contains some common-sense remarks to which parents, schoolmasters, and the medical profession will do well to give attention.

These medical officers adopt the extreme position of neither side; that is, they advocate neither grandmotherly coddling nor the Spartan survival-of-the-fittest attitude of many athletic persons. They point out that neither age nor distance is in any way an exact criterion of the strain inflicted on any given boy by any given race. The quarter-mile, they very justly observe, is a far more exhausting race for most boys than are the long-distance races; and to this we would add the half-mile, in which schoolboys have done at various times very notable performances, but, now and then, with considerable detriment to themselves. It would, in our opinion, be far wiser to prohibit these two distances for boys under eighteen than to interfere with "school runs" and paper-chases. In the latter any chance of a boy damaging himself is practically absent, for the pace set is so much slower than that of flat races that exhaustion, when it does occur, affects the muscles of the legs and thighs rather than of the heart. The Association lays it down that the plan of running all the boys, old and young, over the same distance in these runs, is not to be recommended; but we are not sure that in this particular we quite agree. For if a separation is made it must be on some rough line, such as age; and that means that a compact, well-developed youth capable of any exertion may be sent into the junior division to set the smaller boys a hot pace over a short run, while an overgrown and much less precocious boy a month older may be put to compete with the most athletic of his fellows over the longer distance. When all the boys run together, the best runners may finish a five-mile cross-country run half an hour before the worst; but at the same time this gives those who, by reason of youth or retarded development, cannot excel at this exercise, a chance to complete the run and benefit by it without any undue strain.

Yet another point dealt with by the Association is of vast importance; that is, the relation of meals to active exercise. It is true, of course, that the digestion of the average boy rises superior to disturbing factors which would disorganise that of an adult; but the recommendation not to allow active exercise within an hour of a meal is one with which we agree most emphatically, and one which is disregarded probably at every public school in the kingdom. At regular intervals parents call attention publicly to the spare diet supplied at certain public schools; and we believe that there are many schools at which very serious deficiencies in this respect exist. But in one way a not too liberal midday meal may be no disadvantage; that is, when football or some other active exercise is indulged in immediately afterwards. The Medical Officers of Schools also do good service in pointing out that boxing, rowing, swimming, and long-distance diving are exercises which severely tax adolescents, and before beginning which they should be medically examined just as for running. Another very practical point made is that hockey is a more exhausting game than football. In this they are almost certainly right. Yet another precaution frequently omitted is the avoidance of severe exercise too soon after convalescence from infectious fevers and similar illnesses. Boys are usually so full of repressed energy at such times that they require supervision to prevent excess in this direction.

The situation, then, amounts to this: That the best medical authorities—for so we regard the Association of which Dr. Shelly is president—are agreed that, provided there is a thorough medical examination of every boy on entering school; provided that the effects of various games on the younger boys are carefully supervised; provided that certain common-sense rules, which boys themselves often do not appreciate, are enforced upon them, then the risk of ordinary school exercises, including cross-country runs and flat races not exceeding one mile, is so reduced as to be quite negligible. Over-anxiety of parents about their children is only one of the manifestations of the increasing tendency of the day towards neurasthenic and introspective psychoses. At this time of the year large numbers of boys are turning their energies towards their annual school sports. If the masters and school medical officers do their duty in observing the limits laid down by the Association, parents may safely leave their boys to follow their own inclinations and their school traditions in these matters without fear of the consequences.

